# Angiopoietin-Like Proteins in Angiogenesis, Inflammation and Cancer

**DOI:** 10.3390/ijms19020431

**Published:** 2018-02-01

**Authors:** Carmine Carbone, Geny Piro, Valeria Merz, Francesca Simionato, Raffaela Santoro, Camilla Zecchetto, Giampaolo Tortora, Davide Melisi

**Affiliations:** 1 Digestive Molecular Clinical Oncology Research Unit, Department of Medicine, University of Verona, 37134 Verona, Italy; genypiro@hotmail.com (G.P.); franziskasimio.fs@gmail.com (F.S.); raffaela.santoro@univr.it (R.S.); 2 Laboratory of Oncology and Molecular Therapy, Department of Medicine, University of Verona, 37134 Verona, Italy; giampaolo.tortora@univr.it; 3 Medical Oncology Unit, Azienda Ospedaliera Universitaria Integrata, 37134 Verona, Italy; valeriamerz@gmail.com (V.M.); camilla.zecchetto@gmail.com (C.Z.)

**Keywords:** angiopoietin-like proteins, angiogenesis, inflammation, cancer

## Abstract

Altered expression of secreted factors by tumor cells or cells of the tumor microenvironment is a key event in cancer development and progression. In the last decade, emerging evidences supported the autocrine and paracrine activity of the members of the Angiopoietin-like (ANGPTL) protein family in angiogenesis, inflammation and in the regulation of different steps of carcinogenesis and metastasis development. Thus, ANGPTL proteins become attractive either as prognostic or predictive biomarkers, or as novel target for cancer treatment. Here, we outline the current knowledge about the functions of the ANGPTL proteins in angiogenesis, cancer progression and metastasis. Moreover, we discuss the most recent evidences sustaining their role as prognostic or predictive biomarkers for cancer therapy. Although the role of ANGPTL proteins in cancer has not been fully elucidated, increasing evidence suggest their key effects in the proliferative and invasive properties of cancer cells. Moreover, given the common overexpression of ANGPTL proteins in several aggressive solid tumors, and their role in tumor cells and cells of the tumor microenvironment, the field of research about ANGPTL proteins network may highlight new potential targets for the development of future therapeutic strategies.

## 1. Introduction

Angiopoietin-like proteins are eight secreted glycoproteins showing structural similarity to members of angiopoietin family proteins.

**Figure 1:**
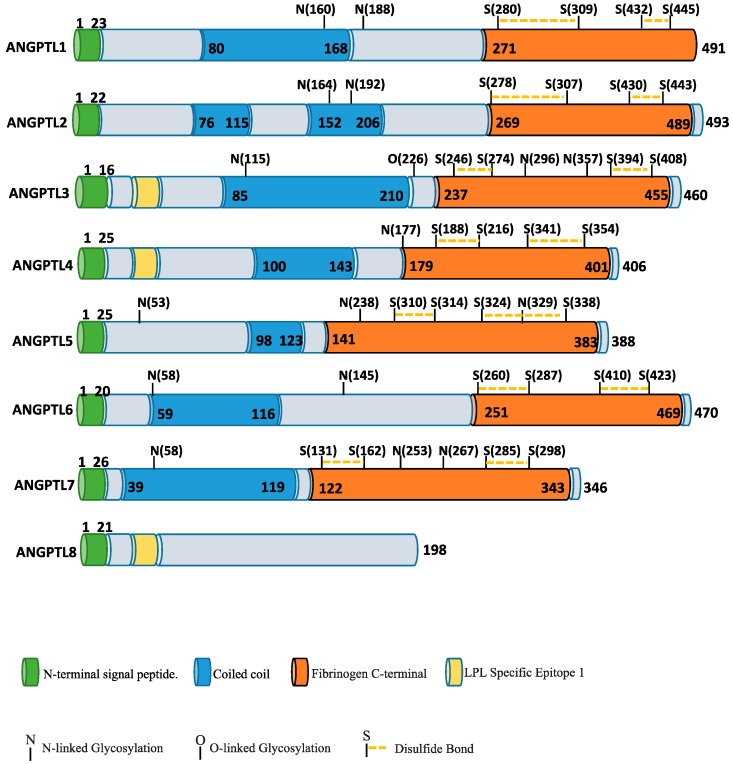
Schematic representation of ANGPTL proteins structure. The ANGPTL family has three conserved domains; a N-terminal signal peptide, a coiled coil domain and a fibrinogen like domain.

Angiopoietins bind Tie1 and Tie2 receptor tyrosine kinase mainly expressed on endothelial and early hematopoietic cells [1]. These receptors are important modulators of angiogenesis and preservation of vascular integrity and permeability [1]. Two domains characterize angiopoietin and angiopoietin-like protein family: an N-terminal coiled-coil domain that mediates homo-oligomerization, a linker peptide, and a C-terminal fibrinogen-like domain, that binds Tie2 (Figure 1). The only exception is ANGPTL8, which has no carboxy-terminal (cANGPTL) domain. Angiopoietin/Tie receptor signaling cascades are involved in fundamental angiogenesis events including vascular stabilization and remodeling, as well as recruitment of pericytes and smooth muscle cells. Although the similarity sequence among angiopoietin and angiopoietin-like proteins [2,3], none of the latter bind to either Tie2 or Tie1, and have been generally considered orphan ligands [4,5].

ANGPTLs are widely expressed in many tissues including liver, vascular system and hematopoietic system and play important roles in inflammation, lipid metabolism and angiogenesis [5].

Leukocyte immunoglogulin-like receptors (LILRs), also called CD85 or immunoglobulin-like transcripts (ILTs), have recently been described as receptors for several angiopoietin-like proteins (ANGPTL-2, -5, -7) [4]. LILRs comprise a family of immunomodulatory molecules that are expressed on human and mouse hematopoietic stem cells [6]. The binding of ANGPTLs to these receptors sustained ex vivo expansion of hematopoietic stem cells and play a role in the maintenance of stemness of normal adult stem cells and in support of cancer development and progression [6]. Oligomerization of ANGPTL proteins via the N-terminal coiled-coil domain is required to trigger LILR signaling. However, although none of ANGPTL proteins bind to the angiopoietin receptors, most members show angiogenic effects [7,8,9] and seems to regulate lipid, glucose, and energy metabolism as well as to promote cancer progression and metastasis [7,10,11,12,13,14].

Here, we review what is currently known about ANGPTL proteins in angiogenesis, inflammation and cancer progression.

## 2. ANGPTL Protein Family and Angiogenesis

The formation of new blood vessels through processes called angiogenesis is extremely important for the proliferation, metastatic spread and resistance of cancer cells. The balance between both angiogenic activators and inhibitors regulates angiogenesis. Up to date, different proteins have been described as angiogenic activators and inhibitors. And their expression levels reflect the aggressiveness of tumor cells.

In the last years, several papers demonstrated the role of ANGPTL protein family in angiogenesis. Here we summarized the last findings on ANGPTL proteins and angiogenesis (see Table 1).

ANGPTL1, also known as angioarrestin, was the first discovered member of the ANGPTL family. It was identified as an anti-angiogenic factor that inhibits endothelial cell proliferation, migration, and adhesion of endothelial cell [15,46]. ANGPTL1 works as a tumor suppressor gene by inhibiting angiogenesis, invasion and metastasis, improving clinical outcomes in hepatocarcinoma (HCC) patients [16]. ANGPTL1 protein inhibits vascular endothelial growth factor (VEGF) and basic fibroblast growth factorf (bFGF)-induced proliferation specifically on endothelial cell types exerting also its antiapoptotic activity through the induction of phosphorylation of extracellular-signal-regulated kinase 1/2 (ERK1/2) and protein kinase B alpha (AKT1) [17]. Thus, ANGPTL1 protein is among the factors whose expression balance angiogenesis and permeability.

ANGPTL2 is an adipose tissue-derived secretory glycoprotein. It is expressed in heart, adipose tissue, kidney, lung and skeletal muscle. Various studies showed that ANGPTL2 plays a role in metabolic syndrome [19], angiogenesis [20] and inflammation [21]. Although several potential receptors has been proposed to link ANGPTL2, such as integrins α5β1 and Toll-like receptor 4 (TLR4), up to date the leukocyte immunoglobulin-like receptor B2 (LILRB2) was the unique receptor known for ANGPTL2 [6]. ANGPTL2 role in angiogenesis was well demonstrated, both in vitro and in vivo experiments [20,22,23,24,25,26].

Recently Oike and colleagues reported two distinct functions for ANGPTL1 and ANGPTL2 by generating transgenic mice expressing these ANGPTL proteins. While ANGPTL1 expressing mice showed a wild-type similar phenotype, ANGPTL2 expressing mice showed a significant increase in the number of blood vessels. Thus, demonstrating that ANGTL2 plays a role as proangiogenic factor, indeed ANGPTL1 negatively regulates angiogenesis. Thus, it has been proposed a model by which ANGPTL1 and ANGPTL2 interact with unknown endothelial cells receptor or receptors to modulate angiogenesis in a context-dependent manner [2,7,8].

Also Kubota Y. and colleagues demonstrated a cooperative interaction between ANGPTL1 and ANGPTL2 proteins. Both proteins likely function as either pro- or antiangiogenic factors, depending upon the context. The single knockdown of ANGPTL1 or ANGPTL2 resulted in normal phenotype in the vascular system during zebrafish embryogenesis. However, the imbalance from this equilibrium state or the knockdown of both ANGPTL1 and ANGPTL2 produced severe vascular defects due to increased apoptosis of endothelial cells at the sprouting stage [47].

ANGPTL3, is a liver-specific secreted factor, mainly involved in the metabolism of triglyceride-rich lipoproteins by inhibiting the activity of lipoprotein lipase (LPL) [48,49,50].

The importance of ANGPTL3 for lipid metabolism was first indicated by the genetic analysis of a mutant strain of obese mice with low plasma lipid levels [51]. ANGPTL3 levels were inversely correlated with very low density lipoprotein (VLDL) and intermediate-density lipoprotein (IDL)—cholesterol levels, and positively with high-density lipoprotein (HDL) cholesterol. Significant correlations have been reported with systolic blood pressure, plasma low density lipoprotein (LDL) and plasma adipocyte fatty acid binding protein (A-FABP) [52].

However, while the N-terminal region of ANGPTL3 has been identified as essential for regulation of plasma triglyceride levels in mice, the C-terminal region containing the fibrinogen-like domain is important for the angiogenic action of ANGPTL3 [27,53].

The receptor for ANGPTL3 is not clear as for LILRB2. The C-terminal domain of ANGPTL3 bounds to integrin receptor alpha-5/beta-3-dependent (α5β3) [27]. This activation was sufficient to induce endothelial cell adhesion and in vivo angiogenesis. The ANGPTL3-integrin-α5β3 binding induced endothelial cell adhesion and migration, and stimulated signal transduction pathways characteristic for integrin activation. Moreover, the combination of ANGPTL3 with growth factors like stem cell factor (SCF), thrombopoietin (TPO), insulin growth factor-2 (IGF-2), fibroblast growth factor-1 (FGF-1) yielded a very significant expansion of stem cells that were able to provide long-term hematopoiesis in mice [54].

Recently, ANGPTL3 was involved in liver cell proliferation and maintenance during zebrafish embryogenesis. The knockdown of ANGPTL3 in the liver zebrafish leads to abnormal liver developing, which is caused by suppression of cell proliferation, but not by enhancement of apoptosis. However, the ANGPTL3 knockdown did not alter angiogenesis in the developing liver [55] 

ANGPTL3 drugs targeting are currently available and have been tested in phase 1 and 2 studies only on metabolic disorders.

In this research field, it has been recently demonstrated that inhibiting ANGPTL3 mRNA expression with antisense oligonucleotides (ASOs) reduced atherosclerosis and levels of atherogenic lipoproteins in mice.

A phase 1 trial recently demonstrated that IONIS-ANGPLT3-LRX, a second-generation ligand-conjugated antisense drug, reduced ANGPTL3 protein and atherogenic lipoproteins levels with no serious adverse events correlated to the treatment [56].

Two phase 2 studies with this drug are ongoing in patients affected by metabolic disorders (NCT03360747, NCT03371355).

Another therapeutic strategy targeting ANGPTL3 has been developed with a monoclonal antibody called evinacumab (REGN1500). In a phase 1 trial evinacumab demonstrated to reduce plasma triglycerides and low-density lipoprotein (LDL) and high-density lipoprotein (HDL) levels. The most frequent adverse events were headache (11%) and elevation of transaminases levels (3%) [57].

Another phase 1 study with evinacumab is ongoing in Caucasian and Japanese healthy volunteers (NCT03146416).

Overall, little data is nowadays available on safety, tolerability, pharmacokinetics, and pharmacodynamics of ANGPTL3 inhibitors, therefore results from ongoing clinical trials will be essential to understand all these aspects and prompt their use in clinical practice.

Like ANGPTL3, also ANGPTL4 seems to have an important role in regulating lipid storage and breakdown [58]. Indeed it is also named fasting-induced adipose factor (FIAF) or hepatic fibrinogen/angiopoietin-related protein (HFARP) or peroxisome proliferator-activated-receptor-γ angiopoietin-related protein (PGAR) [58,59,60]. ANGPTL4 is the best-studied member of ANGPTL proteins family and it exhibits a widespread distribution of tissue expression.

The expression of ANGPTL4 in the liver and adipose tissue is altered after feeding and fasting, suggesting a role for ANGPTL4 in regulating fat metabolism. It inhibits LPL, an enzyme that hydrolyzes triglycerides from the apolipoprotein B, and increases intracellular lipolysis of triglycerides in adipocytes, determining the reduction in adipose tissue weight [61]. ANGPTL4 plasmatic levels decreased by insulin playing a key role in type 2 diabetes mellitus and metabolic syndrome [62,63]. Moreover, ANGPTL4 expression in a transgenic mice model of diabetes appears to improve angiogenesis thus accelerating wound re-epithelialization [64].

ANGPTL4 is also involved in energy homeostasis, redox regulation, inflammation, endothelial cell integrity, angiogenesis and development of cancer [28]. The wide roles of ANGPTL4 are strictly related to its complex protein structure.

ANGPTL4 is cleaved by pro-protein convertases, releasing nANGPTL4 and the C-terminal portion of ANGPTL4 (cANGPTL4) [65].

cANGPTL4 is responsible for these functions independently of nANGPTL4. cANGPTL4 binds and activates integrin 5β1α-mediated Rac1/PAK signaling to weaken cell-cell contacts [66,67].

Although ANGPTL4 is the most studied member of the ANGPTL proteins family, the current role in angiogenesis is still under investigation. It has been reported as pro- or anti-angiogenic protein and proposed to play a role as gatekeeper regulating vascular integrity and angiogenesis in a context-dependent manner. Some studies indicate that ANGPTL4 can protect the integrity of endothelial cells, while others have shown that it can be destructive to the endothelium, thereby leading to the initiation of atherosclerosis [68].

Recently, it has been demonstrated that tumor-derived ANGPTL4 suppressed in vitro vascular tube formation and proliferation of human umbilical vascular endothelial cells, through suppression of ERK signaling [69]. Moreover, ANGPTL4 expression levels were increased by two potent negative modulators of angiogenesis, peroxisome proliferator-activated receptors (PPAR)-γ and -α agonists [70,71]. In addition, transgenic mice overexpressing ANGPTL4 (K14-Angptl4) show no evidence of increased vascularity in skin tissues compared with controls [3].

Conversely, some studies reported that ANGPTL4 exerts a VEGF-independent proangiogenic effect [72]. Some authors hypothesized that conflicting reports of ANGPTL4 activity reflect a difference in extracellular matrix binding capacity between full-length and truncated soluble forms of the protein. Only full-length ANGPTL4 can bind extracellular matrix via heparin/heparin sulfate proteoglycan in vitro. Once bound, ANGPTL4 suppresses formation of actin stress fibers and focal contacts in endothelial cells, thereby blocking adhesion and sprouting [29]. ANGPTL4 tunes endothelial cell junction organization and perycites coverage and controls vascular permeability and angiogenesis, both during development and in pathological conditions [73].

ANGPTL5 is structurally similar to other members of ANGPTL protein family, owning a cleavable signal peptide in N-terminal, a coiled-coil domain, and a fibrinogen-like domain. Like ANGPTL3 and 4 plays a role in lipid and triglyceride metabolism and vertebrate development [38]. The ANGPTL5 receptor is not yet known. ANGPTL5 is mainly expressed in adipose tissue and adult human hearth [38,39]. Up to date, no data are published about its role in angiogenesis.

Likewise, to ANGPTL4 and 5, ANGPTL6 remains an orphan ligand. However, unlike the uncertain role of ANGPTL4 and 5, ANGPTL6 also known as Angiopoietin-related growth factor (AGF) has been directly identified as proangiogenic factor [20], although it has also a key role to regulate energy metabolism in an endocrine manner [40]. Oike and colleagues demonstrated that in vitro recombinant ANGPTL6 protein had a chemoattractive effect on endothelial cells and mice overexpressing ANGPTL6 protein had an increased microvessel density [22,41].

ANGPTL6 is an angiogenic factor involved in epidermal proliferation, wound healing [74,75] and mediates adhesion by interacting with integrin receptors through activation of ERK1/2 and endothelial nitric oxide synthase (eNOS) pathway responsible for the generation of nitric oxide (NO) [2,20].

Forced expression of ANGPTL6 in the skin promoted angiogenesis and epidermal hyperplasia compared to wild-type mice, suggesting that ability of tissue repairing is disjointed from its role as an angiogenic factor in the skin [2].

Likewise other members of ANGPTLs family it is shown that angiopoietin-related growth factor directly regulates lipid, glucose, and energy metabolism independent of angiogenic effects in animal studies. Patients with metabolic syndrome show higher serum ANGPTL6 levels compared with healthy subjects [76,77].

The current knowledge about ANGPTL7 also known as cornea-derived transcript 6 (CDT6), is still limited and biological role is only marginally known.

*ANGPTL7* gene is localized within an intron of gene encoding mammalian target of rapamycin (mTOR) protein at chromosome 1p [78]. Its protein is secreted as 40–50 kDa monomers that form disulfide-linked homotrimers and tetramers via the coiled-coil domain. Originally discovered in human corneal, it was found over-expressed in glaucomatous aqueous humor where it seems to be involved in the regulation of intraocular pressure and in the pathogenesis of glaucoma [79]. *ANGPTL7* gene is expressed in neural tissues, keratoconus cornea, trabecular meshwork, melanotic melanoma and endometrial cancer [78].

It has been demonstrated that ANGPTL7 promotes in vitro angiogenesis by stimulating the proliferation, motility and invasiveness of human differentiated endothelial cells. Moreover, the same authors demonstrated that ANGPTL7 recombinant protein injected as matrigel sponge in mice was able to promote vascularization of the matrigel sponge by inducing angiogenesis thereby accrediting this molecule as a pro-angiogenic factor [42]. Overexpression of ANGPTL7 induces expression of collagen and exerting a pathogenic role in glaucoma [80,81]. Recently, Xiao et al., generating ANGPTL7 knockout mice demonstrated that ANGPTL7 is essential for hematopoietic stem cells repopulation [82].

ANGPTL7 is a potent target gene of WNT/β-catenin signaling pathway; therefore it is considered a potential objective of regenerative medicine and oncology [81].

ANGPTL8, also known as betatrophin, TD26, re-feeding induced fat and liver (RIFL), lipasin or C19orf80, is a novel protein predominantly expressed in human liver [43,44]. It differentiates from other ANGPTL family member because it has no fibrinogen-like domain, glycosylation sites and aminoacids for forming disulfide bonds. ANGPTL8 was initially described as a tumor-associated antigen [45], but subsequent studies positioned its function in lipid metabolism, regulating plasma triglycerides levels. Using immunoprecipitation analysis, it has been demonstrated that human ANGPTL8 interacts with full-length ANGPTL3 and the isolated N-terminal fragment of ANGPTL3. Expression of ANGPTL8 increased plasma levels of triglycerides and non-esterified fatty acids in wild type mice, but not in *Angptl3* knockout mice. Infection of mice with both *ANGPLT8* and *ANGPTL3* dramatically increased plasma triglyceride and fatty acid levels. In cultured HepG2 hepatocytes, ANGPTL8 promoted cleavage and secretion of the functional ANGPTL3 N-terminal fragment. Serum ANGPTL8 levels were low after a 12-h fast in humans and increased significantly within 3 h of feeding regulating postprandial triacylglycerol and fatty acid metabolism through activation of ANGPTL3 [83].

Moreover, ANGPTL8 induces pancreatic β-cell proliferation and insulin release in an insulin-deficient mouse model of insulin resistance [83,84,85].

## 3. ANGPTL Proteins in Inflammation and Cancer

ANGPTL family proteins affect not only proliferation and motility of endothelial cells but also stimulate inflammation and incite tumor cell behavior.

Inflammatory processes play absolutely a pivotal role in carcinogenesis, including mechanisms of initiation, growth, proliferation, invasion, angiogenic switch and metastasis. There are substantial evidences that ANGPTL proteins are involved in various ways in human cancer as mediators of inflammatory carcinogenesis (see Table 2) [13,86,87].

The role of ANGPTL1 in cancer is still little known. It had found to be broadly expressed in adult tissues, with higher expression in adrenal gland, placenta, and small intestine. Down-regulation of ANGPTL1 was demonstrated in kidney, lung, prostate, bladder, and thyroid cancers when compared with non-tumor adjacent tissue. ANGPTL1 can suppress SLUG (SNAIL-related zinc-finger transcription factor) to inhibit cancer cell motility [88]. Like angiogenesis, the balance between activators and inhibitors regulates the progression of tumor growth. Thus, based on ANGPTL1 in vitro activity and on expression profile, it was postulated that ANGPTL1 could play an inhibitory role in tumor progression [17].

ANGPTL1 has anti-angiogenic activity in vitro and tumor suppressive activity in vivo. Effectively, ANGPTL1 overexpression in breast tumor cells resulted in a striking reduction in number and size of tumor nodules, as well as primary melanoma tumors derived from ANGPTL1-secreting cells grow more slowly in vivo compared to empty vector transfected cell [17].

Moreover, results from the ectopic overexpression studies suggest that ANGPTL1 may play an essential role in tumor inhibition by affecting different angiogenic processes necessary for tumor growth. It has been reported that ANGPTL1 treatment significantly inhibited in vitro and in vivo migration and invasion ability of lung cancer cells. Moreover, ANGPTL1 was responsible for reorganization of cytoskeleton through inhibition of actin stress fiber formation resulting in an altered cellular morphology. In addition IHC analysis of lung tumor tissues revealing that patients with higher level of ANGPTL1 have low metastasis as well as longer survival time [89]. More recently TC Kuo and colleagues validated a mechanism by which ANGPTL1 through integrin α1β1, miR-630 and SLUG pathway induces mesenchymal-to-epithelial transition (MET) allowing cancer cells to regain epithelial properties. Thus suggesting that ANGPTL1 inhibits motility and invasiveness of cancer cells [88].

Recently, Chen and colleagues demonstrated that ANGPTL1 inhibits sorafenib resistance and cancer stemness in HCC cells preventing MET with a mechanism similar to lung cancer, proposing, thus, ANGPTL1 as a novel MET receptor inhibitor for advanced HCC therapy [16].

Unlike ANGPTL1, ANGPTL2 is better acknowledged for its adverse pro-inflammatory properties and its contribution in cancer, diabetes, atherosclerosis, metabolic disorders and many other chronic diseases [90,91]. It is primarily secreted by adipose tissues and its expression is increased in obesity and obesity-related pathological conditions, including hypoxia and endoplasmic reticulum (ER) stress [21]. ANGPTL2 plays a key role in inflammation of adipose tissue via inflammatory vascular remodeling and recruitment of macrophages into adipose tissue [19,92,93]. ANGPTL2 acts as an important rheumatoid synovium-derived inflammatory mediator in rheumatoid arthritis (RA) pathogenesis [92]. In fact, the circulating levels were significantly lower in patients with osteoarthritis compared with rheumatoid arthritis. ANGPTL2 was able, in fact, to induce the chemotactic activity of rheumatoid arthritis synovial fluid-derived monocytes/macrophages and endothelial cells, promoting synovial inflammation. Likewise in rheumatoid arthritis, high expression of ANGPTL2 has reported also in dermatomyositis. It has been demonstrated that the expression of ANGPTL2 in the skin genetically engineered mice model was responsible for chronic inflammation through the NF-κB cascade. Finally, in ligamentum flavum ANGPTL2 stimulation promoted NF-κB nuclear translocation and induced IL-6 expression and secretion promoting tissue inflammation [94].

Thus making ANGPTL2 as a new extremely relevant clinical therapeutic target.

The mechanisms by which ANGPTL2 can achieve its functions are still under investigation. Recently, it has been reported that ANGPTL2 could be cleaved and inactivated into domain fragments by the tolloid-like 1 (TLL1) protease [95]. While the N-terminal coiled-coil domain is supposed to form dimeric or trimeric coiled-coils and enhance survival and replicative ability of hematopoietic cells [96], the remaining fibrinogen-like domain is able to bind some putative receptor-like TLR4 [4,19,23]. To date the LILRB2 is the only identified ANGPTL2 receptor [14,97]. It was hypothesized that ANGPTL2 is a target of homeobox (HOX) proteins [98].

The first report of ANGPTL2 as a helper of inflammatory carcinogenesis and metastasis was provided only few years ago [87]. Up to date several studies reported the role of ANGPTL2 in cancer suggesting it as a tumor promoting gene.

In particular some studies investigated the function of ANGPTL2 proteins and their clinical relevance in several oncogenic settings.

ANGPTL2 is able to promote proliferation and invasion in esophageal cancer (EC) cells. In fact, elevate levels of ANGPTL2 in EC tissues correlate with a higher tumour, node and metastasis (TNM) staging classification stage in patients affected by esophageal tumor. The authors thus confirmed an independent prognostic value of ANGPTL2 in patients with high tissue levels of this protein suffered of worse prognosis and earlier recurrence after surgery than those with low tissue protein expression. ANGPTL2 expression could discriminate patients who are more likely candidates to post-operative chemotherapy preventing tumor relapse, regardless of previous treatments. Moreover, circulating ANGPTL2 allowed to distinguish between patients with EC from healthy controls with high diagnostic accuracy (AUC value > 0.9) bracing its diagnostic marker role, as for gastric cancer (GC) and colorectal cancer (CRC).

ANGPTL2 is a potential novel biomarker for gastric cancer. Likewise EC, the serum levels ANGPTL2 in gastric cancer patients were higher than those of healthy controls [99]. Analogue results derived from another study, where ANGPTL2 knockdown inhibited gastric cell proliferation, invasion and migration [100]. On the other hand, ANGPTL2 was overexpressed in gastric cancer tissues and a further upregulation was associated with tumor progression, early recurrence and poor prognosis [101].

Toyama et al. recently showed that tumor microenvironment induces ANGPTL2 expression in colorectal cancer (CRC) and, on the other hand, ANGPTL2 promotes metastatic ability of CRC cell, furthering tumor’s progression through the enhancement of angiogenesis and EMT [102]. In this study, serum ANGPTL2 levels demonstrated high sensitivity (54.2%) and sensibility (94.3%) to distinguish patients with early-stage CRC compared to normal controls. It’s noteworthy how, even with carcinoembryonic antigen (CEA) values within normal range, serum ANGPTL2 could still discriminate CRC patients from healthy controls (AUC values over 0.8, some of the highest AUC levels for serum biomarkers compared with CEA 0.68 and CA 19.9 0.65 [102]. Moreover, higher ANGPTL2 serum levels correlate with more advanced TNM stage (large tumor, serosal invasion, venous invasion, lymph node, liver and peritoneal metastasis) demonstrating to be an independent predictor for tumor recurrence for CRC patients who underwent surgery. The authors also investigated ANGPTL2 mRNA levels in CRC cells finding them significantly increased compared to normal controls and associated with worse disease free survival (DFS) (*p =* 0.019) and overall survival (OS) (*p =* 0.03) among patients with CRC. They finally observed a significant positive correlation between ANGPTL2 expression in CRC lesions and in serum samples (*p =* 0.048). This report supports the hypothesis that serum ANGPLT-2 protein would be more likely secreted by primary CRC tissues and it may represent a noninvasive prognostic and predictive biomarker for screening diagnosis and monitoring of CRC [101].

The predictive and prognostic values of ANGPTL2 in CRC are also supported in a recent study where authors showed that higher expression of ANGPTL2 in primary tumor tissues is related to aggressive phenotype of tumor cells and enhances resistance to antineoplastic drugs through apoptosis antagonism. Furthermore, a lower overall response rate (ORR) in patients who underwent chemotherapy for unresectable CRC was related to higher ANGPTL2 levels in primary tumors. These results highlight possible new approaches to overcome cancer cells’ chemo-resistance by blocking ANGPTL2 signaling [103].

Pancreatic cancer (PC) remains one of the most lethal and poorly understood human malignancies [104,105]. It has the lowest 5-year relative survival rate among solid tumours at 8% and is projected to become the second leading cause of cancer-related death by 2030 in Western countries. Poor prognosis in PC is attributed to its early metastatic behaviour, aggressive clinical course, and limited efficacy of chemotherapeutic treatments [106].

Our research group recently demonstrated that ANGPTL2 is among the proinflammatory factors overexpressed in PC cells leading to EMT and, in turn, acquired resistance to anti-VEGF treatment [107,108]. In a recent pre-clinical study we elucidated the role of ANGPTL2 and its receptor in EMT process and early metastatic behavior of cells in pancreatic preneoplastic [14]. Crucially, the human leukocyte immunoglobulin-like receptor B2 (LILRB2) has been identified as the receptor for ANGPTLs. A deficiency in the signaling of its mouse orthologue paired immunoglobulin-like receptor (PIRB) resulted in increased differentiation of leukemia cells [6]. Moreover, we proved that an autocrine signaling between ANGPTL2 and its receptor LILRB2 was able to induce early EMT and the tumor progression of a model of pre-neoplastic pancreatic ductal cells [14]. These findings support the possible role of ANGPTL2 for early detection, metastasis chemoprevention and combination treatments [109] of PC.

ANGPTL2 expression correlates with intrahepatic metastasis in patients affected by HCC and accelerates potential of HCC cell lines [110] and have also a role in driving metastasis in human lung cancer [13,111].

There are some evidences about how ANGPTL2 promotes breast cancer cells’ recruitment to bone metastatic sites. In pre-clinical models, it has been demonstrated that ANGPTL2 strengthens responsiveness of breast cancer cells to chemokine (C-X-C motif) ligand 12 (CXCL12) by the up-regulation of chemokine (C-X-C motif) chemokine receptor 4 (CXCR4) in those cells [11]. These findings may suggest a predictive role of ANGPTL2 and possibly new therapeutic approaches to treat metastatic breast cancer. Moreover, circulating ANGPTL2 levels match the clinical features of breast cancer progression, raising a possibility that serum ANGPTL2 levels in breast cancer patients could be a potential marker of metastasizing ability [10].

Conversely, ANGPTL2 was found to suppress the growth of ovarian cancer cell and loss of its function is implicated in carcinogenesis of ovarian cancer [112].

In prostate cancer, androgen deprivation increases ANGPTL2 cancer tissue levels leading in turn to androgen-indipendent and malignant behavior of prostatic cancer cells through an autocrine and/or paracrine manner via integrin α5β1 receptor. Moreover, the authors showed that ANGPTL2 is upregulated in human prostate cancer tissues after neoadjuvant hormonal therapy and this result suggests its clinical relevance in the hormone refractory mechanisms of prostate cancer. Targeting ANGPTL2 pathway may be then a novel promising therapeutic approach for prostate cancer, especially in androgen-independent state [113].

The ANGPTL2 protein promotes migration and metastasis of osteosarcoma cells by promoting tumor cell intravasation mediated by the integrin α5β1, p38MAPK, and MMP proteins [95].

The same authors demonstrated that an experimental model of skin cancer, ANGPTL2 induced inflammation and oxidative stress, generating a tumor microenvironment that supports methylation, and consequently reducing gene expression of DNA repair enzymes such as mutS homolog 2 (MSH2), lead to DNA mutations and cancer initiation. Analogously, in squamous carcinoma skin tissues exposed to sun, MSH2 levels were inversely correlated with ANGPTL2 expression [13].

Moreover, ANGPTL2 promotes EMT via activating TGF-β-Smad pathway in a mouse model of skin squamous cell carcinoma (SCC) expressing ANGPTL2 [87].

Because serum ANGPTL2 level increases in different chronic diseases potentially invalidating its cancer correlation, it is unclear if ANGPTL2 expression is actually related to cancer itself or is just a result of the alterations that occur during cancer’s onset and progression arousing by inflammation and changes in immune response [10,92,114].

Few evidences are reported in literature about the ANGPTL3’s role in cancer growth and invasion through MAPK cascade’s activation. The main significance is attributed to its aberrant expression in several types of human cancers and to its role in new blood vessel growth and stimulation [115,116,117,118].

ANGPTL3 is significantly upregulated in tumor cells compared to normal tissues in oral squamous cell carcinoma (OSCC) derived cell lines and in primary tumors.

Moreover, higher ANGPTL3 levels were correlated with advanced TNM stage and worse OS in patients affected by OSCC. In vitro and in mouse models, ANGPTL3 knockdown reduces cancer cell proliferation and growth upregulating cyclin-dependent kinase inhibitors so arresting cell-cycle at G1 phase. These data support the potential role of ANGPTL3 as a diagnostic and prognostic marker and therapeutic target in patients with OSCC [115]. In HCC in vitro cells, ANGPTL3 inhibited cell proliferation and invasion through downregulation of p38MAPK and MMP-9 cascade’s activation [118]. In ovarian cancer the differential expression of ANGPTL3 gene in women with high-grade serous ovarian carcinoma is a prognostic factor [119].

ANGPTL4 is associated to angiogenesis, tumor cell motility and invasiveness [30,31] cell migration [32], endothelial cell function, vascular leakage, neoangiogenesis [3] and cell adhesion and motility interacting with matrix proteins [29,33]. Recently, ANGPTL4 was suggested to be an important player in redox-mediated cancer progression [34,35]. It was also shown to be involved in arthritis [120]. TGF-β regulates the expression of ANGPTL4 via a Smad3-signaling pathway promoting extravasation of tumor cells and their ability to colonizing lung tissue. Thus, ANGPTL4 impairs the integrity of vascular tight junctions and increases capillaries permeability in the lung promoting, in turn, the intravasation into the lung tissue [28].

Recent findings revealed its role in cancer growth and progression, angiogenesis, metabolism and metastasis. The diverse roles of ANGPTL4 in human cancer are mainly attributed to its capability to target different cellular systems, such as cancer microenvironment, endothelial or metabolic cells. Properly to its wide-spectrum of action, the roles of ANGPTL4 in human cancer are still controversial. Moreover, the high grade of complexity was due to the presence of a proteolytic signal by which was generated two isoforms: an N-terminal coiled-coil domain (nANGPTL4), and a large ANG/fibrinogen-like COOH-terminal domain (cANGPTL4). The presence of different ANGPTL4-derived-peptide with tissue-dependent functions suggests that ANGPTL4 may have different roles in human cancers [121]. It has been reported that ANGPTL4 roles may be totally altered depending on its proteolytic cleavage and posttranslational modifications. Whereas the ANGPTL4 N-terminal domain was mainly involved in the endocrine regulatory role of lipid metabolism, insulin sensitivity, and glucose homeostasis, the COOH-terminal fibrinogen-like domain may be a key regulator of the complex signaling during cancer development. The complex mechanisms of action of ANGPTL4 gets to its possible clinical application as a promising candidate for clinical intervention against cancer.

Despite the increasing emphasis on the differential roles of ANGPTL4 full-length, cANGPTL4, and nANGPTL4, the biological significance of each of them is still to be elucidated in cancer. However, is clear that ANGPTL4 posttranslational modification can affect its biological functions complicating further the role of ANGPTL4 in cancer [28]. Moreover, the tumor microenvironment influences the transcriptional regulation of ANGPTL4 in cancer. Elevated expression of ANGPTL4 is widespread in tumors [122].

While high ANGPTL4 expression is correlated with poor prognosis in oral cancer, it seems to inhibit melanoma and lung cancer tumor growth, metastasis and angiogenesis [28].

The roles of ANGPTL4 in human cancers are most likely complex. Overexpression of ANGPTL4 can further increase tumorigenesis, angiogenesis, metastasis but equally decrease vascular permeability, cells’ motility and invasiveness depending on the contexts of different cancers [28,123]. It is clear that ANGPTL4 overexpression induced an elevation of adenylate energy charge by ANGPTL4 enhances EMT [124].

Tan and colleagues attempt to demonstrate the differences in the tumor characteristics and energy metabolism showing that mutational status of ANGPTL4 correlates on its ability to binding to integrin α5β1 leading to weaker activation of downstream signaling. The ANGPTL4 mutant tumors showed a reduced proliferation, anoikis resistance, and migratory capability as well as a reduced adenylate energy charge [36].

The relation between inflammation and tumorigenesis is well known in CRC. In has been demonstrated, in fact, that high expression of cyclooxygenase-2 (COX-2) in the 50% of colorectal adenomas and in up to 85% of adenocarcinomas correlates with poor survival of CRC patients [125]. COX-2 is strongly associated to PGE2, the most abundant prostaglandin found in CRCs. A recent study showed a link between ANGPTL4 and PGE2 in promoting cell proliferation in hypoxic condition in CRC cells.

Specifically, hypoxia induced the expression of EP1, a PGE2 receptor, in CRC, enhancing, in turn, ANGPTL4 expression and cANGPTL4 secretion. The same authors demonstrated that cANGPTL4, and not full-length ANGPTL4 or nANGPTL4, increased CRC cell proliferation and tumor growth both in vitro and in vivo [65,126,127]. In CRC cells, STAT1 induction is dependent on production of superoxide (O_2_^−^) and NADPH-oxidase, Src and mitogen-activated protein kinase (MAPK) signaling. Consistently Zhu and colleagues demonstrated that cANGPTL4 stimulates a redox-based mechanism enhancing tumor cell survival by alteration of the O_2_^−^ to H_2_O_2_ ratio, leading to the activation of Src and extracellular signal-regulated kinase [34].

Because the different roles of ANGPTL4 fragments, more studies to demarcate the different biological functions of cANGPTL4 and nANGPTL4 in cancer progression are urgently required.

One challenge will be the identification of receptor(s) that mediate the ANGPTL4 signaling pathway [128].

Pre-clinical and clinical studies showed that ANGPTL4 is recurrently expressed in human CRC tissues and cell lines. Overexpression of ANGPTL4 promoted cell migration and invasion [66]. In fact, although its expression did not correlates with overall survival, high expression level of ANGPTL4 relates with the depth of tumor invasion and venous invasion [123].

ANGPTL4 seems to have a role in the metastatic process in oral cancer. It was found that ANGPTL4 expression in biopsy specimens was correlated with the presence of lymph node metastasis and it was suggested that it could contribute to OSCC metastasis by stimulating cell invasion [123]. These results were recently confirmed by others study, showing ANGPTL4 as a potential biomarker and therapeutic target for oral squamous cell carcinoma [129].

Indeed, it has been demonstrated that ANGPTL4 contributes to metastasis by stimulating cell invasion in head and neck carcinoma, in esophageal squamous cell carcinoma (ESCC) and oral squamous cell carcinoma (OSCC) patients [130,131,132]. Using a monoclonal antibody against cANGPTL4 (mAb11F6C4), Zhu and colleagues demonstrated that cANGPTL4, but not nANGPTL4, is expressed in major epithelial tumors such as SCC [60].

Some groups reported that ANGPTL4, through its action on both vascular and tumor compartments, prevents the metastatic process by inhibiting vascular activity as well as tumor cell motility and invasiveness [30].

Moreover, ANGPTL4 expression was significantly lower in HCC tissues than in non-tumor tissues. Low expression of AGPTL4 was significantly associated with advanced tumor stage, poor differentiation as well as poor overall and disease free-survival of HCC patients [46].

The clinical relevance of ANGPTL4 in HCC then has yet to be completely explained. For instance, a recent study showed that tumor tissues had lower levels of ANGPTL4 than non-tumor tissues of HCC patients [133]. Conversely, one study demonstrated that serum ANGPTL4 protein is higher in HCC patients than in chronic hepatitis B patients and normal controls, without defining, however, its expression in HCC [134]. Zhu has conversely demonstrated there is an upregulation of ANGPTL4 in HCC compared to normal liver tissue but in this case, only two HCC specimens were studied on tissue arrays [34]. ANGPTL4 expression was able to discriminate chronic hepatitis cases from controls and those HCC cases from chronic hepatitis patients [135].

A Japanese group recently investigated clinical relevance of ANGPTL4 in HCC testing *ANGPTL4* mRNA levels in tumor and non-tumor liver tissues of HCC patients and their correlation to clinical features The results showed that lower expression level of *ANGPTL4* mRNA in cancer tissues of HCC patients was associated with advanced TNM stage, poor differentiation, higher AFP levels, worse DFS after hepatectomy and OS of patients. In preclinical models treatment with Ab-ANGPTL4 had indeed significantly demonstrated to block angiogenesis, motility and metastasis by destroying tumor-favorable microenvironment. ANGPTL4 may have a diagnostic and prognostic role for HCC patients and a new potential biomarker for future target therapies [46].

The aberrant expression of ANGPTL4 was common to different aggressive cancers. It has been demonstrated that ANGPTL4 was expressed at higher levels in the blood of **breast** cancer patients [136] and high expression of ANGPTL4 correlated with a minor disease-free survival of breast cancer young patients [137]. In addition, performing a whole genome expression profile on circulating tumor cells in breast cancer patients, it has been identified a signature of genes, including ANGPTL4 that distinguished the tumor-aggressiveness of the breast cancer [138]. In triple negative breast cancer (TNBC) ANGPTL4 and VEGFA together to heparin-binding epidermal growth factor (HB-EGF) play a pivotal role in the acquisition of tumor aggressiveness regulating tumor angiogenesis [139]. In vitro studies indicated that PPARβ/δ-ANGPTL4 pathway is involved in the regulation of tumor cell invasion and that its pharmacological manipulation by PPARβ/δ strongly inhibits the serum- and transforming growth factor β (TGFβ)-induced invasion of MDA-MB-231 human breast cancer cells [140].

It was shown that downregulation of ANGPTL4 simultaneously with upregulation of fibronectin 1 (FN1) might be implicated in anti-tumor activity of U94, an oncosuppressor, in androgen resistant prostate cancer cell lines [37]. Consistently in an in vitro model of melanoma cells, ANGPTL4 prevents metastasis through inhibition of vascular permeability, tumor cell motility, and invasiveness [30] 

ANGPTL4 was proposed as a diagnostic marker of primary and metastatic clear cell renal-cell carcinoma [141]. Moreover, in renal cell carcinoma, serum level of ANGPTL4 could be a potential biomarker [142]. A recent paper support dual role for ANGPTL4 in urothelial carcinoma progression, either as a tumor suppressor or oncogene, in response to microenvironmental context [143].

The role of ANGPTL5 in cancer is still weak. It has been reported that LILRB2 and ANGPTL2/ANGPTL5 were co-expressed in cancer cell lines and in primary culture of non-small cell lung cancer NSCLC. The co-expression of ILT4 and ANGPTL5 was associated with low non small cell lung cancer (NSCLC) differentiation and lower overall survival rates [144].

ANGPTL6, also known as angiopoietin-related growth factor (AGF), plays a crucial role in the murine vascular development and/or physiological metabolism [41].

Moreover up-regulation of ANGPTL6 by miRNA-128 contributes to glioma and glioblastoma multiforme (GBM) resulting in the proliferation of undifferentiated GBM cells [145]. It has been reported that accumulation of ANGPTL6 in normal vessels of CRC patients could be a system to tag vessels for circulating cancer cells. The interaction between hepatic ANGPTL6 and tumoral α(6) integrin/E-cadherin drives liver homing and colonization by CRC cells. Deregulating interaction between ANGPTL6 and its receptor inhibits liver metastasis. Consistently, co-expression of α(6) integrin and E-cadherin in primary tumors represent a poor prognostic factor for patients with advanced CRC. Furthermore, this group described an angiopoietin-like 6-mimicking peptide capable of interfering with this interaction, thus acting as an antimetastatic compound [146].

ANGPTL7, also known as Cornea-derived transcript 6 (CDT6), reduces tumor growth and abnormal blood vessel formation by inducing fibrosis. ANGPTL7 was initially described as potent target gene of the Wnt/β-catenin pathway [140].

A recent study demonstrated that ANGPTL7 was overexpressed in colon cancer and, more rarely, in breast and ovary cancers, while it seemed to be expressed at basal level in prostate and lung cancer; moreover, the protein was overexpressed in liver metastasis from CRC. Furthermore ANGPTL7 was described as a pro-angiogenic factor, given that it promoted vascularization in vivo in mouse matrigel sponge assay. This is consistent with its ability to stimulate proliferation, motility, invasiveness and capability to form capillary-like networks in human differentiated endothelial cells [42].

## 4. Conclusions

Although ANGPTL proteins family share highly conserved structural homology with that of angiopoietins, which are recognized to be decisive modulators of angiogenesis, they are involved in several different biological processes, and the balance of ANGPTL proteins could regulate angiogenesis, inflammation, apoptosis, metabolism, and cancer progression.

In particular, ANGPTL1, 2, 3, 4 and 6 likely regulate angiogenesis, whereas angiogenic activities have not been attributed to ANGPTL5 and 7. In addition, ANGPTL3, 4, and 6 regulate also lipid and glucose metabolism.

More specifically, ANGPTL1 has been shown to reduce migratory and invasive abilities of different cancer cell lines in vitro and to suppress the EMT.

ANGPTL2 was initially acknowledged for its pro-angiogenic and antiapoptotic abilities. An excess of ANGPTL2 signaling leads to chronic inflammation and pathological tissue remodeling. Furthermore it can be considered a growth factor since it increases survival and expansion of hematopoietic stem and progenitor cells and may contribute to vasculogenesis. Some authors proposed the utility of detection higher serum ANGPTL2 levels in cancer patients, in particular in metastatic breast cancer patients. Indeed, serum ANGPTL2 levels seem to potentially predict diagnosis, recurrence and poor prognosis in human gastric and CRC. Most importantly, ANGPTL2 role as driver of metastases was demonstrated in lung, breast and liver cancer and in osteosarcoma cell lines.

Different studies reported that ANGPTL3 activity is one of the most important factors in cancer growth and invasion, because of the MAPK signaling cascade.

ANGPTL4 has been reported both as a pro- and an anti-angiogenic protein, regulating vascular integrity and angiogenesis in a context-dependent manner suggesting that it might be tumor-type dependent.

The role of ANGPTL5 in cancer is still pale. It seems that co-expression of ILT4 and ANGPTL5 was associated with low NSCLC differentiation and lower overall survival rates.

Interaction between ANGPTL6 and α(6)-integrin/E-cadherin has been shown to contribute to liver homing and colonization of human CRC cells. Furthermore, it has been provided evidence for a correlation between high levels of ANGPTL6 and poor prognosis in patients with metastatic CRC. ANGPTL6 overexpression contributes to proliferation of glioma and GBM.

ANGPTL7 was overexpressed in colon cancer and, more rarely, in breast and ovary cancers, while it seemed to be expressed at basal level in prostate and lung cancer.

ANGPTL8 expression in human cancer has not been reported yet.

## Figures and Tables

**Table 1:** ANGPTL proteins characteristics and main functions.

Protein	Function	Tissue/Organ Expression	Receptor
**ANGPTL1**	anti-angiogenic, permeability, anti-apoptotic [15,16,17]	Thyroid gland, liver, bladder, gallbladder, gastrointestinal tract (no esophagus), adipose tissue and skin	orphan nuclear receptor, site A apolipoprotein (AI) [18]
**ANGPTL2**	Angiogenesis, development of cancer [19,20,21,22,23,24,25,26]	heart, adipose tissue, kidney, lung and skeletal muscle	integrins α5β1 and Toll-like receptor 4 (TLR4), LILRB2 [4,19,23]
**ANGPTL3**	Angiogenesis and lipid metabolism	liver, kidney	alpha-5/beta-3, LILRB2 (weak) [27]
**ANGPTL4**	Angiogenesis (pro- or anti- agiogenic factor), lipid metabolism, glucose metabolism, energy homeostasis, redox regulation, inflammation, endothelial cell integrity, development of cancer [3,28,29,30,31,32,33,34,35]	adipose tissue, liver, kidney, muscle and intestine, ovary, breast skin, testis, kidney urinary bladder, esophagus	fibronectin, vitronectin, integrin β1 and β5 [36,37]
**ANGPTL5**	lipid and triglyceride metabolism [38,39]	adipose tissue and hearth, ovary, testis, skin,	LILRB2 [4]
**ANGPTL6**	Angiogenesis, lipid metabolism, glucose metabolism [20,22,40,41]	Liver, gallbladder, placenta, bone marrow, placenta	orphan of receptor,
**ANGPTL7**	angiogenesis [42]	eye	LILRB2 (weak) [4]
**ANGPTL8**	lipid metabolism [43,44,45]	liver, adipose tissue	orphan of receptor

**Table 2:** ANGPTL proteins in inflammation and cancers.

Name	Role in Cancer	Inflammation	Cancer Disease Association	Function
ANGPTL1	Tumor suppressor	not reported	low expression in kidney, lung, prostate, bladder, thyroid, breast and lung cancers, melanoma and hepatocarcinoma	Reduces migratory and invasive abilities of different cancer cell lines in vitro and to suppress the epithelial to mesenchymal transition (EMT).
ANGPTL2	Tumor promoting	proinflammatory	high expression in esophageal, colorectal, prostate, pancreatic lung, breast and skin cancers, hepatocarinoma	Pro-angiogenic and antiapoptotic abilities. Increase migratory and invasive ability. Driver of metastases was demonstrated in lung, breast and liver cancer and in osteosarcoma cell lines.
ANGPTL3	Tumor promoting	proinflammatory	high expression in oral squamous cell carcinoma, hepatocarcinoma and ovarian cancer	Cancer growth, motility and invasion.
ANGPTL4	Tumor-type dependent	proinflammatory	high expression in lung, colorectal, oral, breast cancers, hepatocarcinoma, oral squamous cell carcinoma,	A pro- and an anti-angiogenic protein, regulating vascular integrity and angiogenesis in a context-dependent manner suggesting that it might be tumor-type dependent.
ANGPTL5	Tumor promoting	proinflammatory	high expression in non-small cell lung cancer	Among its related pathways are Hematopoietic Stem Cell Differentiation Pathways and Lineage-specific Markers. An important paralog of this gene is ANGPTL3.
ANGPTL6	Tumor promoting	proinflammatory	high expression in glioma, glioblastoma multiforme and colorectal cancer	Tumor growth, and metastases driver.
ANGPTL7	Tumor promoting	proinflammatory	high expression in colorectal, lung, breast and ovarian cancers	A pro-angiogenic factor. Stimulates proliferation, motility, invasiveness and capability to form capillary-like networks in human differentiated endothelial cells.
ANGPTL8	not reported	not reported	Breast Angiosarcoma and Breast Sarcoma	Promote proliferation of pancreatic beta cells and increase insulin release in an insulin-deficient mouse model of insulin resistance. Among its related pathways are Metabolism and Lipoprotein metabolism.
